# A Vesicle Superpool Spans Multiple Presynaptic Terminals in Hippocampal Neurons

**DOI:** 10.1016/j.neuron.2010.03.020

**Published:** 2010-04-15

**Authors:** Kevin Staras, Tiago Branco, Jemima J. Burden, Karine Pozo, Kevin Darcy, Vincenzo Marra, Arjuna Ratnayaka, Yukiko Goda

**Affiliations:** 1 School of Life Sciences, University of Sussex, Brighton BN1 9QG, UK; 2 Wolfson Institute for Biomedical Research, University College London, Gower Street, London WC1E 6BT, UK; 3 Department of Neuroscience, Physiology and Pharmacology, University College London, Gower Street, London WC1E 6BT, UK; 4 Medical Research Council Laboratory for Molecular Cell Biology and Cell Biology Unit, University College London, Gower Street, London WC1E 6BT, UK

**Keywords:** SIGNALING, MOLNEURO

## Abstract

Synapse-specific vesicle pools have been widely characterized at central terminals. Here, we demonstrate a vesicle pool that is not confined to a synapse but spans multiple terminals. Using fluorescence imaging, correlative electron microscopy, and modeling of vesicle dynamics, we show that some recycling pool vesicles at synapses form part of a larger vesicle “superpool.” The vesicles within this superpool are highly mobile and are rapidly exchanged between terminals (turnover: ∼4% of total pool/min), significantly changing vesicular composition at synapses over time. In acute hippocampal slices we show that the mobile vesicle pool is also a feature of native brain tissue. We also demonstrate that superpool vesicles are available to synapses during stimulation, providing an extension of the classical recycling pool. Experiments using focal BDNF application suggest the involvement of a local TrkB-receptor-dependent mechanism for synapse-specific regulation of presynaptic vesicle pools through control of vesicle release and capture to or from the extrasynaptic pool.

## Highlights

► Imaging and EM were used to track fate of vesicles tagged in hippocampal synapses ► Vesicles were not confined to one synapse but spanned multiple terminals ► Mobile vesicles were available as an extension of the classical recycling pool ► Vesicle sharing could be locally regulated by BDNF

## Introduction

Presynaptic terminals in hippocampal neurons harbor defined vesicle pools, which are major determinants of synaptic performance (Rizzoli and Betz, 2005; Südhof, 2004). In conventional models of synaptic transmission, these pools are synapse-specific, with vesicles being locally recycled after exocytosis at the same terminal (Ceccarelli et al., 1973; Heuser and Reese, 1973). As such, presynaptic function is characterized by the number and properties of vesicles within an individual terminal. Recent experimental evidence, however, shows that some synaptic vesicles (SVs) can move between adjacent release sites in mature neurons (Chen et al., 2008; Darcy et al., 2006a; Fernandez-Alfonso and Ryan, 2008; Krueger et al., 2003; Westphal et al., 2008), raising the possibility that vesicles arising from outside a synaptic terminal might contribute to its presynaptic function. For example, if vesicles were trafficked at high rates across multiple terminals and were readily available to all neighboring synapses, this would represent a common vesicle pool that could underlie axonal synapse-synapse interactions. To directly test this possibility, we characterized the spatiotemporal organization of vesicle sharing in hippocampal neurons using fluorescence imaging and correlative light and electron microscopy (EM). Our findings, in dissociated cultures and acute hippocampal slices, strongly support the existence of a large vesicle resource or “superpool” composed of some of the recycling pool vesicles from many adjacent terminals that can be rapidly and directly accessed by individual synapses. Such an arrangement provides a unique perspective on presynaptic organization at central terminals.

## Results

### A Vesicle Pool Common to Multiple Synaptic Terminals

**Figure 1:**
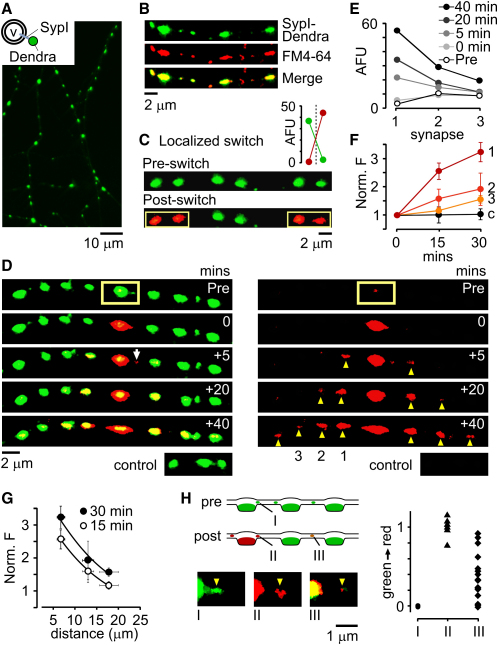
Vesicle Sharing Visualized with a Photoswitchable Fusion Construct (A) SypI-Dendra2 construct (v, vesicle) expressed in hippocampal culture. (B) SypI-Dendra2 colocalizes with FM4-64. (C) Pre- (top) and post- (bottom) photoswitch of SypI-Dendra2 in two synaptic pairs (yellow rectangles). Plot shows mean intensities for red and green fluorescence before and after photoswitch. (D) Photoswitch of a single synapse (yellow rectangle) to examine long-range vesicle traffic with composite of green and red fluorescence (left) and red fluorescence only (right). A discrete mobile packet leaves the photoswitched synapse (arrow) and red fluorescence accumulates at neighboring synapses (arrowheads). Bottom panels: control synapses within the same field of view but on different processes to a switched bouton. (E) Quantification of red fluorescence spread in (D). (F) Summary plot of red fluorescence accumulation over time normalized to starting value for the three synapses neighboring a switched source bouton (n = 7). (G) Summary plot of data in (F) showing red fluorescence against distance from switched synapse. (H) Analysis of vesicle packet types: mobilized directly from unswitched synapse before photoswitch (“pure green,” I), mobilized directly from newly switched synapse measured at first time point after photoswitch (“pure red,” II), and mobilized at sites remote from the switched synapse at up to 9 min after the photoswitch (III). Bottom: examples of different packet types. Right: summary plot of green-red composition for packet types I, II, and III on a normalized scale of their red:green ratio (pure green = 0, pure red = 1).

Studies characterizing lateral vesicle traffic (Chen et al., 2008; Darcy et al., 2006a; Fernandez-Alfonso and Ryan, 2008; Hopf et al., 2002; Krueger et al., 2003; Westphal et al., 2008) have mainly relied on single-color vesicle markers, but these probes offer limited information about the origins and fates of mobile vesicles across multiple synapses over time (Figure S1 available online). To explore spatiotemporal dynamics of SV traffic in detail, we designed a vesicle probe using a photoswitchable fluorochrome, Dendra2, which can be rapidly and irreversibly photoswitched from a green- to a red-emitting form following brief intense exposure to 488 nm light (Gurskaya et al., 2006). We fused Dendra2 to the C terminus of Synaptophysin I (Takamori et al., 2006) and expressed the resulting fusion protein (SypI-Dendra2) in hippocampal cultures (Figure 1A). SypI-Dendra2 showed punctate distribution that colocalized with the activity-dependent vesicle marker FM4-64 (Figures 1A and 1B) and was closely apposed to the postsynaptic marker PSD-95 and the dendritic marker MAP2 (Figure S2), confirming its expression at functional presynaptic terminals. Focal 488 nm laser illumination selectively photoswitched synapses in the target area, with typically a >40-fold increase in red fluorescence intensity and a 12-fold decrease in green fluorescence intensity (Figure 1C).

Localized photoswitching of SypI-Dendra2 was used to “tag” vesicles at a synapse along an unbranched length of axon, and the movement of new red fluorescence to adjacent regions was monitored to examine the contribution made by individual synapses to the mobile vesicle population over time. Immediately after photoswitching, red signal was confined to the switched synapse, but over time it spread widely as discrete mobile packets (white arrow, Figures 1D and 1E) and accumulated at boutons that were often spatially remote from the source synapse (>30 μm), separated by multiple terminals. Also, green fluorescence reaccumulated at the source synapse, consistent with turnover of switched red signal with unswitched green signal originating from synaptic neighbors (Figure 1D). We quantified red fluorescence spread for all experiments by measuring red fluorescence intensity at the three flanking synapses on each side of the source synapse at 0, 15, and 30 min after photoswitching (Figure 1F). The extent of accumulation of red signal at synapses along an axon was directly related to the distance of the synapse from the source bouton (Figures 1F and 1G). Importantly, synapses within the same field of view, but not sharing the same axon as the switched bouton, did not accumulate red fluorescence (Figures 1D and 1F), indicating that the gradual appearance of red signal at synapses was not caused by a nonspecific photoswitch process, but rather resulted from vesicle movement between boutons sharing the same axon. Thus, vesicles from individual synapses are not restricted from sharing with adjacent neighbors, but instead are rapidly distributed across many widely separated boutons. For the whole population of synapses along an axon, mobile vesicles therefore form a significant vesicle resource or superpool that is commonly accessible to multiple synaptic terminals.

Next, we asked whether vesicle redistribution to remote terminals involved multiple local exchange events or direct movement between spatially discrete synapses, bypassing intermediate terminals. Analysis of SypI-Dendra2 packets at interbouton regions separated from a source synapse by one or more unswitched terminals revealed different vesicular compositions, from pure green through to pure red (Figure 1H, type III). This suggests that transiting packets can readily acquire vesicles from synapses or intersynaptic regions to form new mobile units with variable vesicular compositions. However, examples of red packets at distant sites also imply that mobile vesicles can skip stable synaptic terminals and pass directly to remote synapses while retaining their original vesicular identity. Thus, the shared vesicle pool spans multiple synapses, with traveling vesicle packets being directly accessible to a population of synaptic terminals.

### Ultrastructural View of the Vesicle Superpool

**Figure 2:**
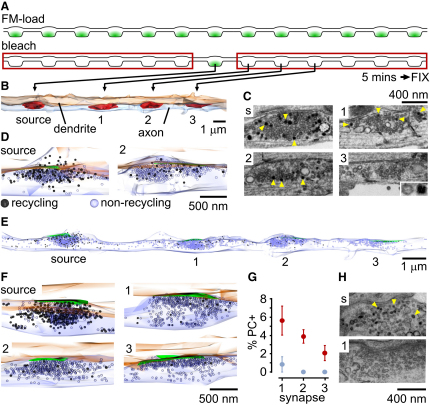
Ultrastructural Readout of Functional Vesicle Sharing from a Target Synapse (A) Experimental scheme. (B) Ultrastructural reconstruction of target process showing axon, dendrite, and SV clusters (red). (C) Sample EM images from synapses in (B), fixed after ∼5 min. Top left (“s”): unbleached source synapse. Recycling vesicles (PC+) have dark lumen (arrowheads) and nonrecycling vesicles (PC−) have clear lumen, which are readily distinguishable (inset). (D) Reconstruction of vesicle clusters from “source” synapse and synapse “2” from (B). Green, active zone. (E) Full reconstruction of axon and vesicles from (B). (F) A second example illustrating lateral spread of recycling vesicles arising from the synaptic source into bleached synapses. (G) Summary of vesicle sharing from a source terminal to synaptic neighbors showing PC+ vesicles as a percentage of total vesicle count for each synapse (1, 2, 3) adjacent to an unbleached source synapse, 0 (blue) or 5 min (red) after photobleaching. Intersynaptic distances were not significantly different for control (5.4 ± 0.2 μm) versus experimental conditions (5.5 ± 0.5 μm) (t test, p > 0.92). Values are mean ± SEM. (H) Sample EM images of synapses from 0 min control group: unbleached synapse (top) and adjacent photobleached synapse (bottom).

SypI-Dendra2 provides an informative readout of vesicle sharing dynamics but offers a restricted view of the detailed organization of the shared vesicle pool. For example, conventional light microscopy limits the visualization of mobile vesicle traffic to large and clustered vesicle packets. It is not clear whether such vesicle modules reflect the true organization of the shared vesicle pool or if single vesicles could also be mobilized between boutons. Also, SypI-Dendra2 does not discriminate between functionally active vesicles and those in the nonrecycling pool, even though the vesicle dynamics may be dependent on the functional class of vesicles or their recent history. To address these issues directly, we employed a correlative fluorescence and EM method to examine properties of the shared pool in ultrastructural detail (Darcy et al., 2006a, 2006b). The total recycling pool in synaptic terminals was labeled with a fixable form of FM1-43 dye (Betz and Bewick, 1992; Ryan et al., 1993). Single axonal processes with multiple sequential FM-dye-labeled synapses were identified and subjected to a reverse FRAP protocol (Figure 2A) in which fluorescence of a single target synapse was preserved while flanking terminals were rapidly photobleached. Neurons were fixed after 5 min, FM-dye was photoconverted (Darcy et al., 2006a; Harata et al., 2001; Rizzoli and Betz, 2004; Schikorski and Stevens, 2001), and samples were processed for serial section EM. In this way, recycling vesicles contributed by a single target bouton to the neighboring regions over 5 min could be visualized and quantified. As controls, target terminals were photobleached and fixed immediately.

In an axon fixed after 5 min, the target (unbleached) synapse contained both photoconverted (PC+) and nonphotoconverted (PC−) vesicles (Figures 2B–2D), representing recycling and nonrecycling vesicles, respectively. The average fraction of PC+ vesicles was 40.4% ± 7.2% of the total pool at target terminals (n = 4, Figure 2D). Notably, PC+ recycling vesicles were also present in flanking synapses, with the highest proportions at terminals adjacent to the target synapse, and the lowest at more distally located terminals (Figures 2E–2G, see also Figure S3). In control experiments where cultures were fixed immediately after bleaching, the unbleached target synapses contained a higher proportion of PC+ vesicles (54.9% ± 9.6%, n = 4 synapses), and neighboring bleached terminals contained essentially no PC+ vesicles (Figures 2G and 2H). Thus, the bleach protocol was sufficient to prevent the subsequent photoconversion of FM-dye-labeled vesicles. Taken together, these results suggest that PC+ vesicles accumulate at photobleached synapses by lateral trafficking (∼3%–5% of total pool/min) from a single nonphotobleached “source” synapse, indicating that individual synapses distribute functionally recycling vesicles to a wide synaptic neighborhood over time. Serially reconstructed axons also highlight the appearance of the shared vesicle pool, with vesicles typically distributed across much of the intersynaptic span (Figures 2E and S3). Some vesicles are arranged in tight clusters of large vesicle packets, but others are less contiguous or present as single vesicles. Overall, vesicles at areas between synapses (average separation: 4.85 ± 0.43 μm) represent a substantial fraction (11.9% ± 2.8%, n = 10 intersynaptic regions) of the average total vesicle pool at flanking synapses.

Next we examined if all or a subset of recycling vesicles at a terminal belong to the laterally mobile pool. Single presynaptic terminals (n = 9) in FM-dye-loaded neurons were photobleached and, after a 1 hr recovery period, prepared for ultrastructural analysis as above (Figure S4). Whereas large numbers of new recycling vesicles were seen at photobleached synapses after 1 hr compared to numbers in newly bleached control terminals (n = 6, Figure S4), these still represented a subset (∼40%) of the total recycling pool that we measured at unbleached synapses after the same 1 hr period (n = 4, Figure S4). Given the high rate of vesicle mobility we observe over short timescales, the incomplete longer-term recovery suggests that recycling vesicle pools may include both highly mobile and more stable (i.e., those likely to be retained) vesicle fractions, implying a possible heterogeneity in whether vesicles are associated with (or belong to) the superpool.

### Vesicle Sharing in Native Hippocampal Tissue

**Figure 3:**
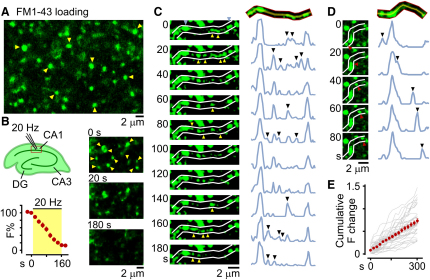
Lateral Sharing of Recycling Vesicles in Native Hippocampal Tissue (A) FM1-43-labeled synapses (examples shown with arrowheads) in CA1 region imaged using two-photon microscopy. (B) Top left: schematic. Right: destaining of FM puncta (arrowheads) by local 20 Hz stimulation at 0, 20, and 180 s. Bottom left: plot showing stimulation-evoked fluorescence loss for 26 puncta. (C) Sample time-lapse sequence (left) and corresponding line scan plots (right) showing multiple trafficking events (arrowheads) along an axon between stable puncta (red arrows). (D) A discrete trafficking event in which fluorescent packet (arrowhead) passes through a stable terminal. (E) Cumulative fluorescence intensity change plot for n = 39 boutons.

To date, the characterization of intersynaptic vesicle movement has been limited to work in cultured neurons (Chen et al., 2008; Darcy et al., 2006a; Fernandez-Alfonso and Ryan, 2008; Krueger et al., 2003; Westphal et al., 2008), and the relevance of this phenomenon to presynaptic organization in native tissue remains unclear. We addressed this question in acute hippocampal slices using two-photon microscopy to image presynaptic terminals labeled with FM1-43. After dye-loading, we observed discrete fluorescent puncta corresponding to presynaptic terminals in region CA1 as reported previously (Zakharenko et al., 2001) (Figure 3A). These labeled terminals were release competent because their fluorescence destained upon stimulation (Figure 3B). Axonal regions between stable puncta showed bidirectional trafficking of many fluorescent packets, large and small, with both merging and shedding events (Figures 3C and 3D), analogous to vesicle movement in culture (Figure S1) (Darcy et al., 2006a). To quantify vesicle flux at stable synapses, we monitored changes in fluorescence levels of single terminals over time. The cumulative fluorescence change corrected for imaging noise shows a linear profile (n = 39: Figure 3E), indicating that at most synapses fluorescence intensity fluctuates continuously, implying a constant vesicle flux through terminals. Our findings strongly support the idea that a shared pool of functional vesicles is a feature of native hippocampal tissue.

### A Shared Vesicle Pool as an Extension of the Recycling Pool

**Figure 4:**
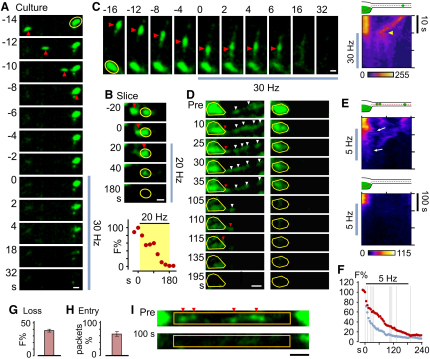
The Shared Vesicle Pool as an Extension of the Recycling Pool at Synapses (A) Example of FM-dye-labeled mobile vesicle packet in culture entering a stable terminal and rapidly undergoing stimulus-driven fluorescence destaining. (B) Top: stimulus-evoked destaining of a synapse (oval) in an acute slice, immediately after incorporation of a mobile vesicle packet. Bottom: destaining plot for the oval region. (C) Fusion capability of an FM-dye-labeled mobile vesicle packet trafficking along an axon segment. Kymograph (right) of a line scan along the axon and synapse (top schematic) shows rapid stimulus-driven fluorescence loss (arrowhead) that does not involve movement into an adjacent presynaptic terminal. (D) Examples of FM-dye fluorescence loss at individual synapses during 5 Hz stimulation. Left: mobile packets (white arrowheads) move into the synapse over time (red arrowheads) and destain. Right: an axon with low vesicle traffic with no mobile packets entering the synapse during stimulation. (E) Kymographs of line scans for synapses in (D). Draining of mobile vesicles from the axon into the presynaptic terminal is seen as diagonal lines of fluorescence (top: arrows). (F) Destaining curves for synapses in (D) (red, left; blue, right). Dashed lines correspond to time points shown in (D). (G) Extent fluorescence loss after 100 s of stimulation along 23 axon segments containing only mobile packets, relative to intensity before stimulation and corrected for photobleaching (from 18 control axon segments). (H) Relative extent of mobile packets along axon segments that moved into the adjacent synaptic terminal. (I) Sample axon segment (rectangles) used for analysis in (G) and (H) before and after stimulation. For comparison the bottom frame is corrected for imaging-related photobleaching. Scale bars, 1 μm. Plots are mean ± SEM.

Mobile populations of extrasynaptic vesicles that are adjacent to stable presynaptic terminals might serve as additional vesicle reservoirs for presynaptic release. While previous work has shown that mobile vesicles enter synaptic terminals and undergo fusion alongside native vesicles (Darcy et al., 2006a), whether incorporation and fusion are sufficiently rapid to contribute to release during sustained transmission has not been considered. We investigated this issue in culture using FM-dye-loaded neurons combined with field stimulation. Mobile vesicles that became newly incorporated into terminals could readily participate in vesicle fusion (Figure 4A). A similar observation was also made in an acute slice preparation (Figure 4B). Complementary to this idea of rapid fusion-competence, we also observed examples of mobile vesicle clusters that underwent FM-dye loss while moving (Figure 4C). Next, we examined the consequence of synaptic incorporation of mobile vesicles during continuous stimulation. A synapse along a process with high vesicle mobility continually received new consignments of fluorescent vesicles that, during stimulation, were released alongside native vesicles (Figure 4D, left). This lateral draining of mobile vesicles into stable synapses can be observed directly in a kymograph plot (Figure 4E), and in this example, resulted in a delayed stimulation-evoked FM-dye loss compared with a synapse on a process that showed low levels of mobile vesicle traffic (Figures 4E and 4F). Quantifying the fate of mobile vesicle packets during activity by measuring stimulus-evoked fluorescence changes in intersynaptic axonal segments (n = 23 from three cultures) revealed a net loss of FM-dye fluorescence signal (39% ± 2.2%: Figure 4G and 4I). This indicates substantial activity-dependent fusion of mobile vesicles originating from axonal regions. To establish what fraction of the packets destained after moving into neighboring terminals (versus those released while moving along the axon), we tracked the fate of the mobile axonal packets prior to their destaining and found that the majority (65% ± 7%, n = 23) of packets entered a flanking synapse during destaining (Figure 4H). Taken together, these findings show that the shared pool of functional vesicles can provide an additional vesicle reserve available to synapses during ongoing transmission.

### Local Regulation of Vesicle Capture/Release at Individual Synapses

**Figure 5:**
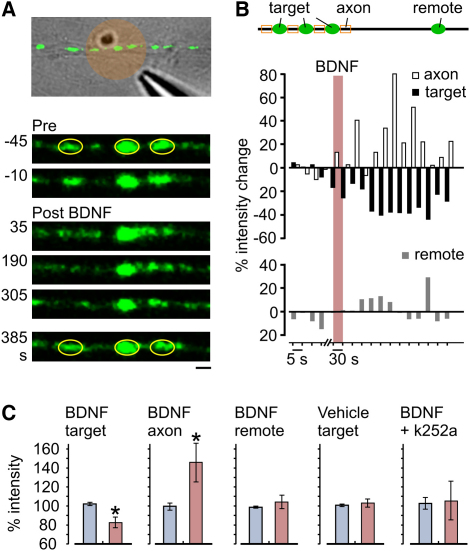
Local Regulation of Vesicle Capture/Release (A) Top: brightfield and fluorescence overlay image showing local BDNF application (circle, estimated local concentration: 200 ng/ml) to SypI-GFP-expressing synapses. Bottom: time-lapse frames showing initially stable boutons (ovals) becoming transiently destabilized after BDNF application before reclustering (385 s). Scale bar, 1 μm. (B) Quantification of fluorescence changes after BDNF for the example in (A), showing percent fluorescence change at three target synapses (black bars), flanking axons (white bars), and a nontarget region (“remote;” see top schematic). (C) Summary of relative fluorescence intensity for pre- (blue bars) and post- (red bars: 5.5 min) BDNF application for five different experimental conditions (n = 5, 5, 5, 4, and 3). ^∗^p < 0.05.

The functional extrasynaptic pool might be particularly relevant for synapse operation if terminals could individually regulate vesicle capture/release processes. BDNF, which plays key roles in synaptic plasticity and remodeling (Lu et al., 2008; Poo, 2001) is a candidate modulator of such events. Previous studies in cultured hippocampal neurons have implicated BDNF-TrkB receptor signaling in controlling SV clustering at terminals via disruption of cadherin-catenin adhesion complexes (Bamji et al., 2003, 2006) and in activity-dependent rapid functional maturation of presynaptic terminals via Cdc42 signaling and actin remodeling (Shen et al., 2006). While in these studies, BDNF signaling has been manipulated globally, local BDNF-dependent regulation of presynaptic scaffold or the cytomatrix could offer a possible mechanism to achieve synapse-specific control of vesicle exchange between individual presynaptic terminals and the vesicle superpool. To test this, we focally applied BDNF onto target synapses in SypI-GFP-expressing neurons and monitored fluorescence over time (Figure 5). Consistent with previous findings, vesicle clusters at target synapses were disrupted following BDNF exposure as evidenced by a reduction in fluorescence intensity compared with that of control synapses that received focal application of vehicle. Moreover, fluorescence in flanking axonal regions increased concurrently, implying that vesicles liberated from target synapses entered the extrasynaptic pool. The observed decrease in synaptic SypI-GFP signal required BDNF-TrkB receptor signaling because it was prevented by pretreatment with the TrkB receptor tyrosine kinase inhibitor k252 (0.5 μM). The effect of BDNF was spatially confined, and synapses on the same process but distant from the site of focal application did not show equivalent declustering (Figures 5B and 5C). This effect was also transient, with SypI-GFP fluorescence intensity at target synapses returning to their initial levels at >7 min after BDNF treatment (fluorescence intensity versus vehicle control: p = 0.62). Thus, control of vesicle release and capture acting via local regulation of BDNF-TrkB receptor signaling provides a possible synapse-specific control mechanism to modulate synaptic size and performance.

## Discussion

In this study, we have used methods to tag vesicle pools in hippocampal synapses and show that vesicles originating from individual terminals are redistributed across a wide synaptic neighborhood. Our findings indicate that synapses can contribute ∼4% of their total vesicle pool/min to extrasynaptic sites and that these trafficking vesicles can enter spatially remote terminals either directly or indirectly. We suggest that some recycling vesicles at individual synapses form a subset of a large shared vesicle resource, or superpool, that spans multiple release sites, an idea previously hypothesized but without definitive experimental support (Staras, 2007; Westphal et al., 2008). We also show that this common vesicle pool is not a culture-specific phenomenon but rather a feature of native hippocampal neurons, thus broadening its potential physiological relevance for presynaptic function to intact neural circuits. Furthermore, we illustrate how vesicles arising from the superpool can enter synapses during stimulation, providing, in effect, an extension of the available vesicle recycling pool at individual terminals. Using focal BDNF application, we demonstrate a potential BDNF-TrkB receptor signaling mechanism for locally regulating vesicle release and capture at individual terminals that permits synapse-specific modulation of vesicle pool sizes.

The existence of a dynamic mobile vesicle pool that is not limited to the boundaries of a presynaptic terminal but instead shared across multiple spatially remote synapses represents a unique view of presynaptic organization. To further explore this idea we constructed a stochastic model of vesicle sharing in which vesicles could be exchanged between both the recycling pool and the adjacent extrasynaptic pools, and directly between neighboring extrasynaptic pools (Figure S5A). Pool sizes and rates of vesicle gain/loss were experimentally derived, and the vesicle “spread” was simulated for one axonal branch with 20 synapses using a range of different vesicle exchange rates between extrasynaptic pools to determine the best match to our ultrastructural data (Figure S5). Our model shows that a given vesicle forming part of the mobile fraction (estimated at 40% of total recycling pool/synapse) will redistribute to a different recycling pool with an average timescale of ∼15 min. As such, mobile vesicles can readily access a wide range of synaptic terminals over short time periods, directly supporting the idea of a superpool. Distant synaptic neighbors are thus coupled through the sharing of a common resource, and this could underlie a variety of synapse-synapse interactions. Notably, our findings correlate well with those from recent studies examining the redistribution of synaptic proteins in mature neurons, both presynaptically (Frischknecht et al., 2008; Kalla et al., 2006; Li and Murthy, 2001; Star et al., 2005; Tsuriel et al., 2006, 2009) and postsynaptically (Ashby et al., 2006; Bannai et al., 2009; Ehlers et al., 2007; Frischknecht et al., 2009; Gray et al., 2006; Okabe et al., 2001; Sharma et al., 2006; Tsuriel et al., 2006) where constitutive, as well as activity-dependent, sharing across multiple synapses in mature neurons has been reported.

What is the overall scale of the superpool? To place a value on this, we first estimated the total synapse numbers per neuron and per axonal branch for our cultures (375 ± 70 and 23.1 ± 0.5, respectively, n = 3) (Figure S5). Assuming an average mobile pool of ∼40% of total recycling pool (Figure S4), and an average recycling pool of ∼195 vesicles, we estimate that ∼1800 recycling vesicles are available for exchange within a local branch. If we include vesicles already residing at extrasynaptic sites (∼12% of the recycling pool at a synapse), this value approaches 2000 vesicles, representing a branch-specific superpool of as much as 10 times the size of the recycling pool at any one synapse. However, because vesicles can move through branch points, it is reasonable to assume that the superpool could extend over multiple branches. In this case, the upper limit on the superpool would be the total estimated number of mobile recycling vesicles (∼33,000), or ∼170 times the size of the recycling pool at one synapse, and time would be the limiting factor for defining the overall scale of the superpool. Our model, in agreement with our experimental data, shows that substantial exchange can occur over ∼20 synapses (or approximately one branch length) over 1 hr.

We also considered the consequences of an extrasynaptic pool for presynaptic operation during sustained release. Previous work has shown that mobile vesicles can enter a terminal and undergo fusion at a later time (∼15 min, Darcy et al., 2006a). Whether this release capability is immediate or acquired gradually has remained unclear, although this bears on the extent to which dynamic vesicle traffic could contribute to ongoing synaptic transmission. Here we provide evidence that mobile vesicles can rapidly attain fusion capability upon entering a presynaptic terminal. Moreover, some mobile vesicle packets are fusion competent while in transit along axons. This extends a previous report of the fusion capability of recently mobile orphan synapses (Krueger et al., 2003) by demonstrating that trafficking vesicles can move and destain simultaneously. What determines whether an individual vesicle packet enters into a synapse or fuses at axonal regions remains unclear, although the former seems to predominate (Figures 4F–4H). Together these observations imply that mobile vesicles could be of considerable relevance to presynaptic terminals during sustained transmission, providing an additional functional SV reserve that extends beyond the conventional boundaries of the synapse.

One key aspect of vesicle sharing is its potential importance for regulating presynaptic performance over time. For example, given that populations of vesicles can have different release modes (e.g., Fredj and Burrone, 2009; Goda and Stevens, 1994; Sara et al., 2005; Sun et al., 2007), vesicles might be functionally heterogeneous. The trafficking of vesicles across multiple synaptic neighbors would provide a means for reallocating functionally distinct vesicles to specific terminals, and could represent a potential mechanism for achieving rapid changes in synaptic properties. Since vesicle redistribution occurs quite rapidly, vesicle sharing could also be relevant for modulating synaptic weights through the resizing of SV pools. Given that release probability (*p_r_*) is known to be directly correlated with recycling pool size (Murthy et al., 1997), changes in the regulatory mechanisms that control the size of SV pools (see below) could therefore profoundly affect synaptic performance. The fact that synapses draw on a pool of shared vesicles from a wide synaptic neighborhood, a substantial fraction of which lie outside the boundaries of a presynaptic terminal, suggests that such resizing of an SV pool at a single synapse could be readily achieved without significantly impacting individual adjacent synaptic neighbors. Such a rapid mechanism for presynaptic strength adjustments could participate in the fast synapse-specific homeostatic changes in *p_r_* and synaptic pool sizes observed in hippocampal synapses (Branco et al., 2008), and in turn, changes in rates of vesicle flux at individual terminals could contribute to intersynaptic variability of *p_r_* (Branco and Staras, 2009; Branco et al., 2009).

How compatible is the wide-scale sharing of vesicular resources with the established concept of synapse specificity? In our experiments only a subset of the total vesicle pool is laterally mobile, suggesting that the identity and specificity of individual synapses can still be preserved. Also, we would favor the argument that synapse specificity is conferred mainly by stable, structural elements of the presynaptic terminal, which also govern the size of SV pools at individual synapses. In support of this, bassoon, an active zone scaffold protein, is very stable and exchanged between boutons over a timescale of hours (Tsuriel et al., 2009). Furthermore, impairing key structural/scaffolding protein complexes of the synaptic junction, such as the cadherin-catenin complex or the MALS proteins, perturbs presynaptic organization by reducing the size of vesicle clusters (Bamji et al., 2003, 2006; Olsen et al., 2005). In this study we demonstrate how vesicle clusters at presynaptic terminals can be directly and individually regulated by focal application of BDNF, a known modulator of vesicle pool organization and release at synapses (Bamji et al., 2003, 2006; Shen et al., 2006; Tyler et al., 2006). Such synapse-specific regulation provides a mechanism to control release/capture of vesicles at individual boutons and thus could play a role in maintaining and/or modulating individual presynaptic terminals. An additional level of regulation could be provided by postsynaptic targets, acting through either structural components or other retrograde messengers to shape presynaptic properties according to the state of postsynaptic activity (Branco et al., 2008; Futai et al., 2007; Regehr et al., 2009). We believe, therefore, that presynaptic differences will be preserved in spite of vesicle sharing and that the idea of a vesicle superpool is not in general conflict with the idea of synapse specificity.

## Experimental Procedures

Full methods are available in the Supplemental Information.

## Supplemental Information

Document S1. Supplemental Experimental Procedures and Figures
