# Supplementary material for: A Vesicle Superpool Spans Multiple Presynaptic Terminals in Hippocampal Neurons
Source: Neuron. 2010 Apr 15;66(1):37–44. doi: 10.1016/j.neuron.2010.03.020 (PMC2908741; doi:10.1016/j.neuron.2010.03.020)
Supplement: Document S1. Supplemental Experimental Procedures and Figures [file mmc1.pdf]

Neuron, volume *66*

Supplemental Information

A Vesicle Superpool Spans Multiple Presynaptic Terminals in Hippocampal Neurons

Kevin Staras, Tiago Branco, Jemima J. Burden, Karine Pozo, Kevin Darcy, Vincenzo Marra, Arjuna Ratnayaka, and Yukiko Goda

Supplemental Experimental Procedures

Preparations

Dissociated hippocampal cultures were prepared from P0 rats as described previously (Darcy et al., 2006a; Morales et al., 2000) and used for experiments at 12-18 days in vitro (DIV). Neurons were transfected at DIV8-9, using a Ca^{2+} phosphate protocol. Experiments were performed in HEPES-buffered bath solution (137 mM NaCl, 5 mM KCl, 2.5 mM $CaCl_2$, 1 mM $MgCl_2$, 10 mM D-Glucose, 5 mM HEPES, 20 μM 6-cyano-7-nitroquinoxaline-2,3-dione (CNQX), 50 μM d(-)-2-amino-5-phosphonovaleric acid (AP5)). Acute brain slices (300 μm) were prepared from 3 wk Sprague-Dawley rats in accordance with national animal care guidelines. Slices were maintained at 32 - 35°C in artificial cerebrospinal fluid (ACSF) containing (in mM): NaCl 125, KCl 2.5, glucose 25, NaH_2PO_4 1.25, $NaHCO_3$ 25, $MgCl_2$ 1, $CaCl_2$ 2, CNQX, 0.02, AP5 0.05 (pH 7.3 when bubbled with 95% O_2 and 5% CO_2). Labeling, imaging and EM methods are detailed in Supplemental Data.

Labelling and imaging in cultured neurons

For cultured neurons, recycling synaptic vesicles were labelled using field stimulation (600 APs 10 Hz) in the presence of the dye (FM4-64 or a fixable analog of FM1-43, FM1-43FX, 10 μM, Molecular Probes, see below). After loading, cells were washed for 1 min in Advasep-7 (1 mM, Biotium Inc.) and subsequently rinsed with fresh bath solution for 10 min to remove surface dye. Plasmid encoding SypI-Dendra2 was constructed by replacing the GFP sequence in SypI-EGFP construct (Darcy et al., 2006a) with that of Dendra2 from the pDendra2-N vector (Evrogen, Moscow, Russia). Dendra and FM-dye FRAP experiments were carried out on a confocal laser microsope (BioRad Radiance 2100) with a 60x 1.0NA dipping objective (Nikon). 512 x 512 images were collected with

the pinhole aperture open. For FM1-43FX experiments, images were acquired with a 488 nm argon laser and a 528/50 filter set. Parameters for photobleaching were consistent with those used in our previous work. We have previously demonstrated that this level of photobleaching does not compromise synaptic vesicle recycling (Darcy et al., 2006a). For SypI-Dendra2, green fluorescence was excited with 488 nm laser light and collected by a 515/30 filter and red fluorescence with 543 nm laser light and 590/70 nm emission. Photoswitching used spatially restricted laser illumination (~ 2 x 2 μm). Quantification of fluorescence was carried out using ImageJ (NIH) on raw unfiltered images or after filtering (1 x 1 median filter) applied to the whole image.

FM1-43 photoconversion and electron microscopy

FM1-43FX labelled cultures were fixed (2% paraformaldehyde / 2% glutaraldehyde, 15 min), washed with 100 mM glycine (1 h), then 100 mM NH_4Cl (5 min) and finally rinsed in phosphate-buffered saline (PBS). For photoconversion, cells were pre-incubated in diaminobenzidine (DAB, 1 mg/ml, DAKO) in PBS (10 min). Target regions from fluorescence experiments were re-identified and brightfield images acquired using an Olympus BX50WI upright microscope. FM1-43FX was photoconverted by illumination with 475/40 nm light from a mercury lamp for 12 min in fresh DAB solution. Neurons were rinsed in PBS, osmicated, stained with tannic acid, dehydrated stepwise in ethanol and embedded in EPON (TAAB) as previously described (Darcy et al., 2006b). Serial sections of embedded neurons were placed on formvar coated slot grids. Samples were viewed using a Phillips EM420 electron microscope. Images were acquired with a 1392 x 1040 cooled CCD camera (Roper Scientific, Inc.). Target boutons were identified by aligning electron micrographs with brightfield images of the same area. For analysis we aligned digital images of serial sections from our process of interest using multiple

membrane and organelle landmarks. 3-d reconstructions were built in Reconstruct (Synapse Web, Kristen M. Harris, http://synapses.clm.utexas.edu/) and rendered using Blender (Blender Foundation). Quantification of vesicles as PC+ or PC- was carried out as described previously (Darcy et al., 2006a).

FM-dye imaging in acute slices

Synapses were fluorescently labeled by washing slices into modified saline containing 40 mM KCl and 10 μM FM1-43 (45 s) following a preincubation in ACSF containing 10 μM FM1-43 (1 min). After loading, slices were rinsed again in the preincubation solution to allow completion of endocytosis. Surface FM-dye was removed by washing the slice into ACSF containing 1 mM Advasep-7, followed by continuous perfusion in fresh ACSF solution (30 min). Two-photon imaging was carried out with a Ti:sapphire pulsed laser (MaiTai, Spectra-Physics) tuned to 900 nm on an Olympus BX51WI microscope (60x objective, 0.9 NA; Olympus, Melville, NY). Synapses were imaged at a depth of approximately 50 - 100 μm from the top surface of the slice in region CA1. Time lapse frames were taken every 20 s, each one generated from a maximal projection of five z-sections (0.5 μm separation) in turn averaged from three separate images. For quantification of vesicle flux through synapses we generated a cumulative fluorescence change plot for synaptic terminals imaged in timelapse sequences, subtracting baseline imaging noise measured using a pollen grain imaged with the same acquisition settings. All data was acquired using custom written software in Matlab 7.2 (Mathworks). Quantification of fluorescence was carried out using ImageJ (NIH) on raw unfiltered images or after filtering (1 x 1 median filter) applied to the whole image.

Modeling

For the data shown in Figure S5, a total of 20 synaptic and 20 extrasynaptic compartments were simulated at ten second iterations. For simplicity, synaptic pools corresponded to the mobile fraction of the recycling pool, and non-mobile recycling vesicles and reserve pool vesicles were not simulated. For each iteration, vesicles were randomly moved: 1) from synaptic to the adjacent extrasynaptic pools, 2) from extrasynaptic to the adjacent synaptic pools and 3) between extrasynaptic pools. The direction of movement was randomized for each vesicle. The initial size of individual synaptic pools was 150 vesicles, and probabilities for 1 and 2 were taken from the experimentally measured rates of vesicle gain and loss. Before each experimental setting, the system was allowed to reach steady state, yielding an average synaptic pool size of 100, and an average extrasynaptic pool size of 50 vesicles. To estimate the rate of vesicle movement between extrasynaptic pools, the probability (p) of this event was gradually increased from 0 to 1, and the best fit to the data in Figure 2G selected (see Figure S5A, best fit was with p = 0.5 - 0.6, average of 50 trials). This rate was then used to produce the data in Figure S5B,C. All simulations were performed in Matlab 7.2 (Mathworks).

Electrophysiology

For the data shown in Figure S5D,E, whole-cell recordings were established as described in Branco et. al (2008), and standard extracellular solution containing FM1-43 (10 µM), CNQX (20 µM) and APV (50 µM) was perfused for ~2 min. Dye loading was started after 30 s with 50 APs at 1 Hz in voltage-recording mode, and dye-free solution was re-perfused 30 s after the end of the stimulation.

Defining the number of synapses available to the superpool

Single neurons were FM-dye labelled via stimulation (50 APs, 1 Hz) through a patch pipette, which also contained Alexa 594. In this way synaptic loading was restricted to the target neuron and the axonal processes could be directly visualized at the same time to count the synapses.

Supplemental References

Branco, T., Staras, K., Darcy, K.J., and Goda, Y. (2008). Local dendritic activity sets release probability at hippocampal synapses. Neuron *59*, 475-485.

Darcy, K.J., Staras, K., Collinson, L.M., and Goda, Y. (2006a). Constitutive sharing of recycling synaptic vesicles between presynaptic boutons. Nat Neurosci *9*, 315-321.

Darcy, K.J., Staras, K., Collinson, L.M., and Goda, Y. (2006b). An ultrastructural readout of fluorescence recovery afer photobleaching using correlative light and electron microscopy. Nat Protoc *1*, 988-994.

Morales, M., Colicos, M.A., and Goda, Y. (2000). Actin-dependent regulation of neurotransmitter release at central synapses. Neuron *27*, 539-550.

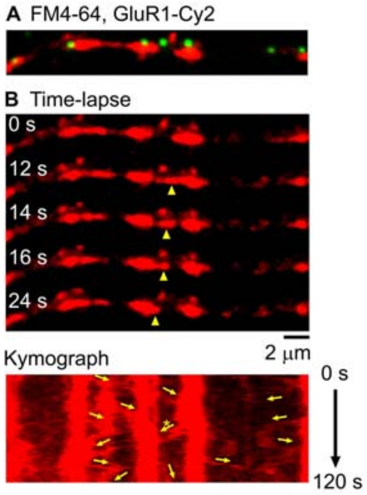

Figure S1. Conventional fluorescent vesicle markers provide limited information on vesicle dynamics. (A) Presynaptic terminals labelled with activity-dependent FM styryl dye, FM4-64 (red) apposed to a postsynaptic antibody label GluR1-Cy2 (green). Live glutamate receptor labelling was carried out as outlined previously (Darcy et al., 2006a). (B) Lateral movement of fluorescent packet between stable terminals (arrowheads) is clearly observed in a time-lapse sequence (top), but longer-term dynamics of individual packets are difficult to follow even in kymographs (bottom). Yellow arrows indicate clear examples of lateral trading events. Asterisk marks the movement event in time-lapse sequence in (B).

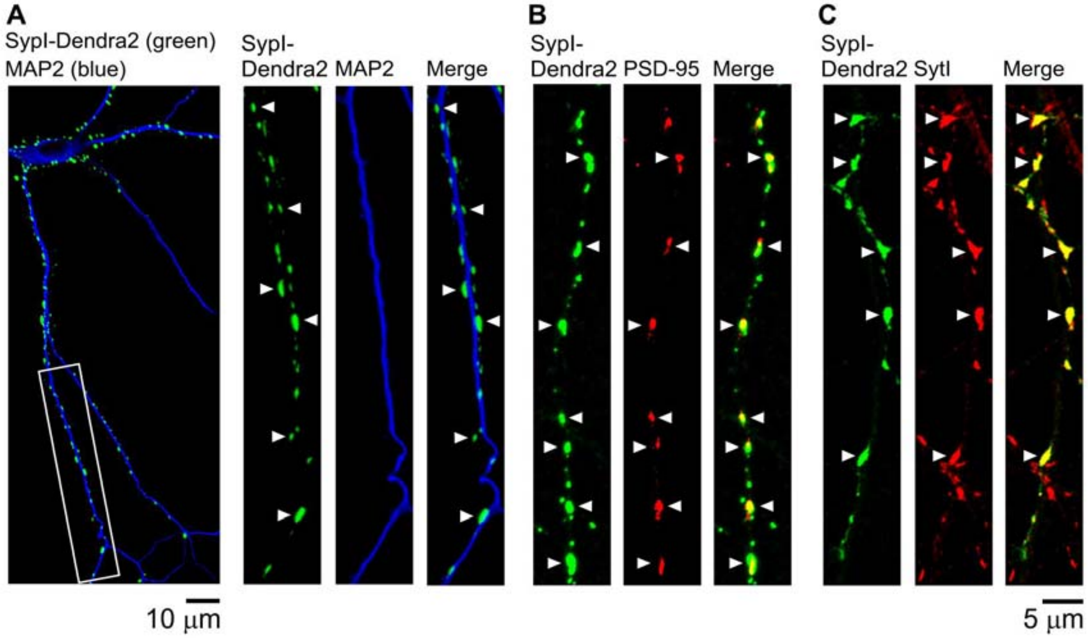

Figure S2. Immunohistochemical confirmation of targeting of SypI-Dendra2 to presynaptic terminals. (A) (left panel) SypI-Dendra2-expressing (green) process (DIV 13) with MAP2 staining (blue). (right panels) Detail of boxed area in left panel showing punctate SypI-Dendra2 expression, MAP2 staining, and merged image. Arrowheads indicate individual examples of SypI-Dendra2 fluorescent puncta closely apposed to MAP2. (B) SypI-Dendra2-expressing puncta apposed to the postsynaptic marker PSD-95. Arrowheads indicate clear examples of apposition. (C) SypI-Dendra2-expressing synapses (green) with Synaptotagmin I (red) staining (Syt I) directed against lumenal domain. Arrowheads indicate examples of clear colocalization of SypI-Dendra2 and Syt I.

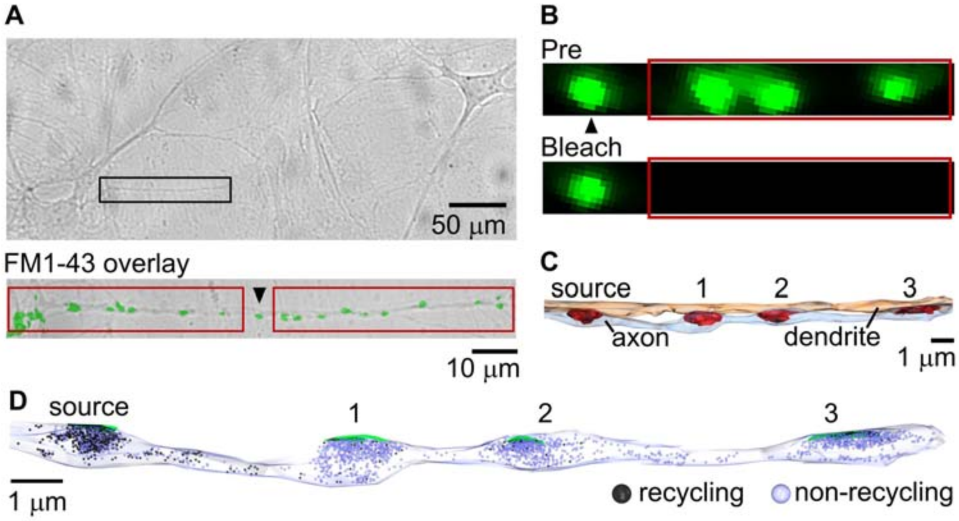

Figure S3. Detail of correlative ultrastructural FRAP experiment. Process is same as in Figure 2F. (A) DIC image (top) shows area of target process (rectangle). (Bottom) overlay of brightfield and fluorescence image showing FM1-43FX staining. Arrowhead shows target synapse and rectangles are photobleach regions. (B) Detail on photobleaching experiment, showing target terminal (arrowhead) and three synaptic neighbours before (top) and immediately after (bottom) photobleaching of area in red rectangles. (C) Ultrastructural reconstruction of target process showing axon, dendrite and main vesicle clusters (red). (D) Full reconstruction of axonal process and all vesicles. Lateral movement of recycling vesicles from 'source' synapse to multiple neighbouring terminals is readily apparent. Vesicles are also seen at intersynaptic sites.

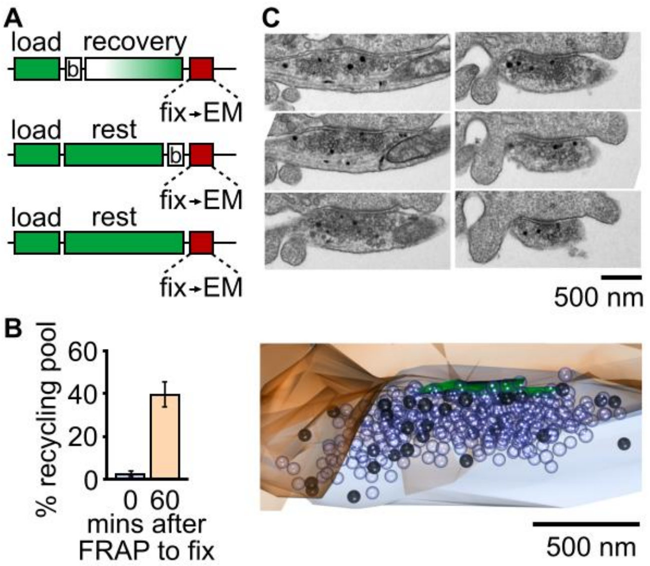

Figure S4. Vesicle mobility is limited to a subset of the total recycling pool. (A) Schematic of experiment. Synaptic recycling pools were loaded with FM1-43FX by field stimulation (600 AP, 10 Hz). For the experimental group (top), target terminals were photobleached (‘b’) and then left for 60 mins in 0.5 mM Ca^{2+} saline before being fixed, photoconverted and prepared for ultrastructural analysis Control bleached synapses (middle) were photobleached (‘b’) after 60 mins in 0.5 mM Ca^{2+} saline and fixed immediately. The recycling pool fraction was determined using a third group of unbleached synapses which were loaded and fixed after 60 mins (bottom). (B) Histogram showing mean ± sem recovery of PC+ vesicles at target synapses (expressed as a fraction of the recycling pool measured at unbleached control synapses) for 0 min and 60 min. At 60 min, recovery in the experimental group is ~40% of the total recycling pool. The recycling pool fraction in unbleached controls was not significantly different to that in unbleached immediate fix controls from the experiments in Fig. 2 (t-test, $p > 0.36$, $n = 4$) suggesting that spontaneous release over 1h did not have a significant impact on our measure of the total recycling pool. (C) (Top) six consecutive serial sections for representative experimental synapse showing new incorporation of mobile recycling vesicles (dark lumen). (Bottom) full reconstruction of same synapse (top).

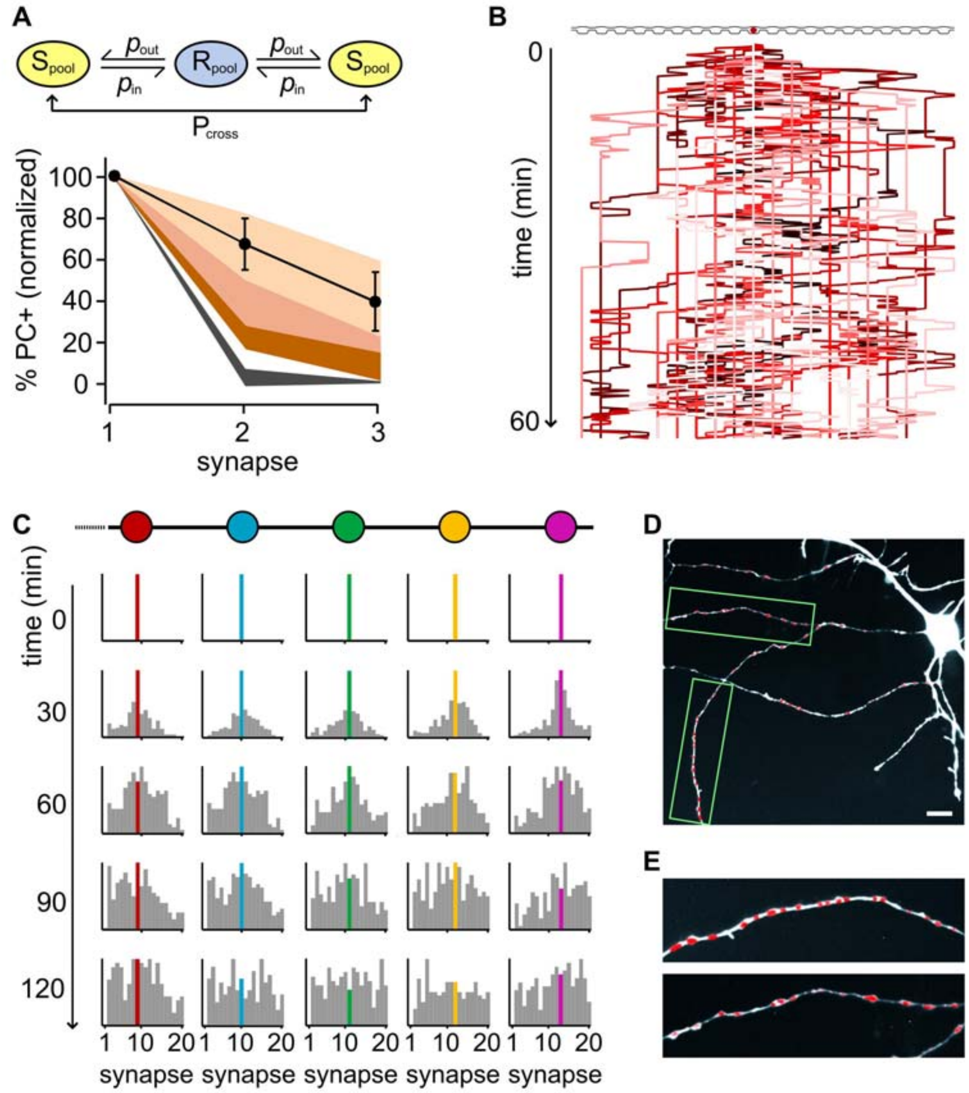

Figure S5. Stochastic model of the vesicle superpool and estimation of total pool size. (A) (Top) schematic illustrating the model compartments: R_{pool}, recycling sharing vesicles residing at the synapse; S_{pool}, extra-synaptic vesicle compartments flanking synaptic terminal. Vesicles move between compartments with probabilities p_{out}, p_{in} and p_{cross} (see Supplemental Procedures for details). (Bottom) simulation of vesicle spread from a source to three synaptic neighbours (1, 2 and 3), equivalent to experiment in Figure 2G, for three vesicle exchange rates between extra-synaptic pools (coloured bands show mean ± SD, pink: p_{cross} = 0.5, brown: p_{cross} = 0.1, grey: p_{cross} = 0). Note pink and brown bands overlap. Circles and error bars are experimentally measured data points for vesicle sharing from the EM experiments (Figure 2G) normalized to the first

synapse. (B) Lateral mobility of a single vesicle derived from a source synapse (50 trials) based on the experimentally-derived parameters. Over a period of 1 h, vesicles can reach and become integrated in any of the neighbouring 19 synapses. (C) Histograms revealing the change in vesicular composition over time for the mobile fraction of recycling vesicles. (Top) middle five synapses of a row of 20 terminals along an axon. (Bottom) plots of vesicles contributed from the synaptic terminals 1 to 20 for each of the five coloured synapses over time. At time 0 (top row), each synapse is filled only with its own vesicles (coloured bars) but over time, vesicles are contributed by synaptic neighbours (grey bars) until the composition of the synapse is substantially changed (bottom row). For clarity, each plot is normalized to the maximum vesicle count. (D) Estimating superpool size by counting synapses. Example image showing overlay of Alexa 594 dye fill (white) and FM-dye synaptic label (red) in a neuron used for these measurements. FM-dye labelling was achieved via stimulation (50 APs, 1 Hz) through a patch pipette, which also contained Alexa. In this way synaptic loading was restricted to the target neuron and the axonal processes could be directly visualized at the same time. Scale bar, 5 µm. (E) Regions of neuron from D (rectangles).
